# Intracranial subdural hematoma after epidural anesthesia: a case report and review of the literature

**DOI:** 10.1186/s12245-018-0199-2

**Published:** 2018-09-06

**Authors:** Victor Szeto, Justin Kosirog, Wesley Eilbert

**Affiliations:** Department of Emergency Medicine, University of Illinois College of Medicine, 1819 West Polk St., Room 469 COME, Chicago, IL 60612 USA

**Keywords:** Subdural hematoma, Epidural anesthesia, Post dural puncture headache, Spinal anesthesia, Postpartum headache

## Abstract

**Background:**

Intracranial subdural hematoma occurring as a result of a procedure that causes a puncture of the spinal dura mater is extremely rare, with less than 100 cases reported. Often, this condition is initially misdiagnosed and treated as a post dural puncture headache.

**Case presentation:**

A woman presented to our emergency department complaining of a headache 4 days after receiving epidural anesthesia during uncomplicated childbirth. The headache’s characteristics were consistent with a post dural puncture headache, and the patient was initially treated as such. Computed tomography later revealed the presence of bilateral intracranial subdural hematomas. In light of the patient’s clinical status, treatment involved cautious observation only. Repeat imaging revealed spontaneous resolution of the hematomas, and the patient had a benign clinical course.

**Conclusions:**

Headaches are common in the postpartum period, often after receiving epidural or spinal anesthesia. While exceptionally rare, intracranial subdural hematoma may occur as a complication of any procedure that results in spinal dural puncture. The possibility of this potentially life-threatening complication must be kept in mind when evaluating these patients.

## Background

First described in 1921, epidural anesthesia involves the injection of anesthetic solution into the epidural space of the spine [1]. It offers the advantages of avoiding general anesthesia and allowing patients to remain awake during surgical procedures. It has become especially popular for obstetric procedures and childbirth. Unintentional dural puncture during epidural anesthesia is not uncommon, occurring with an estimated frequency of up to 3.6% [2]. Intentional dural puncture occurs with spinal anesthesia, where anesthetic is injected into the subarachnoid space. Spinal anesthesia offers the same advantages as epidural anesthesia, with a shorter time of onset [3]. Approximately one third of patients who have had dural puncture will develop a post dural puncture headache (PDPH) [4, 5].

In this report, we describe the extremely rare occurrence of an intracranial subdural hematoma (ISH) following the administration of epidural anesthesia. To our knowledge, this has been reported fewer than 100 times before. In this case, the patient was initially misdiagnosed as having a PDPH.

## Case presentation

A 31-year-old woman with no significant past medical history presented to our emergency department complaining of a constant headache for the previous 4 days. The headache had begun approximately 6 h after receiving epidural anesthesia for labor. The documentation from the anesthesia service that day reported the use of a 17-gauge Touhy needle to enter the subdural space in the lower lumbar spine and the placement of a 19-gauge epidural catheter. No complications were reported with the procedure, and specifically, there was no mention of inadvertent dural puncture. The patient had an unremarkable delivery of a healthy infant at 38-weeks gestation later that day.

**Fig. 1:**
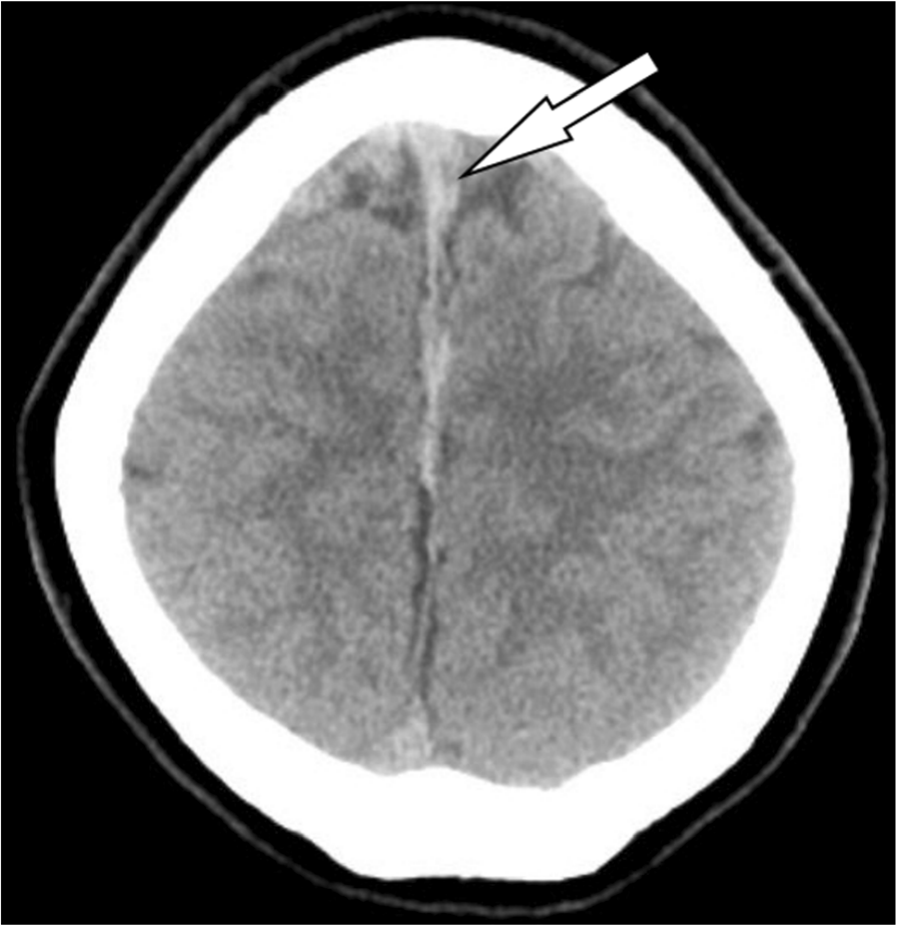
Computerized tomography of the head showing bilateral parafalcine subdural hematomas (arrow)

The patient described the headache as constant and occipital with some radiation to the frontal area. The headache was worse when upright and partially relieved in the supine position. She reported taking acetaminophen/butalbital/caffeine and ibuprofen with little relief. She had no associated vomiting, fever, or changes in her hearing or vision. She denied any photophobia or focal weakness or numbness. She was afebrile on physical exam, with pulse and blood pressure within the normal range. Her exam was notable for a normal neurologic exam including cranial nerves and no neck stiffness. The patient was tentatively diagnosed with a PDPH. After evaluation by the anesthesia service, she was admitted for pain control and possible placement of an epidural blood patch the next day. A computed tomography (CT) scan of her head was obtained prior to admission to evaluate for other possible causes of the headache (Fig. 1). This CT identified bilateral parafalcine subdural hematomas measuring 7 mm in thickness on the left and 3 mm thickness on the right. There was no associated mass effect.

The patient was admitted to the intensive care unit and started on levetiracetam for seizure prophylaxis. Neurosurgical consultation advised observation, and a repeat CT scan of the head the next day showed no significant change in the hematomas. The patient also received an epidural blood patch the next day with no improvement in the headache. A head CT performed on hospital day 3 showed a decrease in the size of the hematomas, and the patient was discharged on levetiracetam for seizure prophylaxis for 1 week.

### Discussion

ISH occurring after dural puncture is extremely rare. Only sporadic case reports and a few small case series have described this condition [6–10]. Any procedure that results in spinal dural puncture will theoretically predispose to the development of an ISH. ISH has been described following epidural and spinal anesthesia, as well as lumbar puncture, myelography, epidural steroid injection, and after implantation of an intrathecal drug delivery device and a spinal cord stimulator [11–15]. The incidence of ISH specifically caused by epidural anesthesia used in obstetric practice has been estimated to be 1:500,000 [16].

The same mechanism has been postulated for both PDPH and ISH [7]. The leakage of cerebral spinal fluid (CSF) from the dural puncture site may continue for several weeks, causing reduction in CSF volume [10]. This results in lower intraspinal and intracranial pressure, leading to relative ventricular collapse and caudal movement of the spinal cord and brain. As a consequence, the dura, pain-sensitive structures, cranial nerves, and subdural bridging veins are stretched. This may ultimately result in a tear of the bridging veins and consequently an ISH. Risk factors associated with ISH after dural puncture include excessive CSF leakage from multiple punctures in large needle use, pregnancy, coagulopathy, cerebral vascular abnormalities, dehydration, brain atrophy, and alcoholism [17–20].

The duration of time from dural puncture to the diagnosis of ISH ranges widely from 4 h to 29 weeks [10]. In one case series, 37% of cases were diagnosed within 1 week of dural puncture, and 85% were diagnosed within 1 month [9]. A headache, most often diagnosed as PDPH, is the main presenting symptom [6, 8–10]. Other reported symptoms and signs present at the time of diagnosis are listed in Table 1 [6, 9, 10]. Reported rates of surgical intervention for ISH after dural puncture vary from 9 to 80% [6, 7, 9]. In general, surgical intervention for ISH is indicated if the hematoma thickness exceeds 10 mm, there is a midline shift of greater than 5 mm, or there is neurologic deterioration [21]. Furthermore, some have advocated for the use of epidural blood patching in the treatment of ISH caused by dural tears resulting in chronic CSF leaks [22, 23]. A full recovery is reported in over 80% of patients, with death reported in 7–10% of cases [7–10].

**Table 1:** Reported symptoms and signs with intracranial subdural hematoma caused by dural puncture

Symptom/sign	Occurrence rate
Headache	74–91%
Nausea/vomiting	31–41%
Altered mental status	31–40%
Focal motor deficit	23–28%
Diplopia/visual changes	14–20%
Aphasia/dysarthria	11–13%

Headache in the postpartum period is common, occurring in 39% of women [24]. The majority of these headaches are benign primary headaches, such as migraine and tension type [25]. Secondary headaches in the postpartum period are typically due to obstetric or anesthetic complications, or the hypercoagulable state after delivery (Table 2). Our patient was initially misdiagnosed as having PDPH, similar to many previous reports of this condition. PDPH is defined as a headache that develops within 5 days of dural puncture that significantly worsens soon after sitting upright or standing and improves after lying horizontally [26]. PDPH is more likely to occur in young women of low body mass as compared with other patients [5]. An epidural blood patch is considered the gold standard for treatment of PDPH, with a success rate of 70–90% [27]. Over 85% of patients report resolution of PDPH within 6 weeks regardless of treatment [9].

**Table 2:** Causes of headache in the postpartum period

Primary headache	Migraine
	Tension type
	Cluster
Secondary headache	Post dural puncture headache
	Pre-eclampsia/eclampsia
	Cerebral venous sinus thrombosis
	Reversible cerebral vasoconstriction syndrome
	Pituitary mass/hemorrhage

The incidence of ISH after dural puncture is probably underreported since many of these patients are treated as PDPH with the eventual resolution of their symptoms. When to obtain brain imaging studies in the assessment of a likely PDPH is unclear. A reasonable approach would be to consider imaging in patients that (1) have a postural headache lasting more than 1 week, (2) do not improve or have worsening of their headache after an epidural blood patch, (3) report a change in the headache from postural to non-postural, or (4) develop other neurologic signs or symptoms with the headache [10].

## Conclusion

Headaches occur commonly in the postpartum period, often after receiving epidural or spinal anesthesia. While exceptionally rare, ISH may occur as a complication of any procedure that results in dural puncture. The possibility of an ISH must be kept in mind when evaluating these patients.

## Abbreviations

CSF: Cerebral spinal fluid
CT: Computed tomography
ISH: Intracranial subdural hematoma
PDPH: Post dural puncture headache
